# Supplementary figures and images for: Pathogenic mutations in the chromokinesin KIF22 disrupt anaphase chromosome segregation
Source: eLife. 2022 Jun 22;11:e78653. doi: 10.7554/eLife.78653 (PMC9302971; doi:10.7554/eLife.78653)

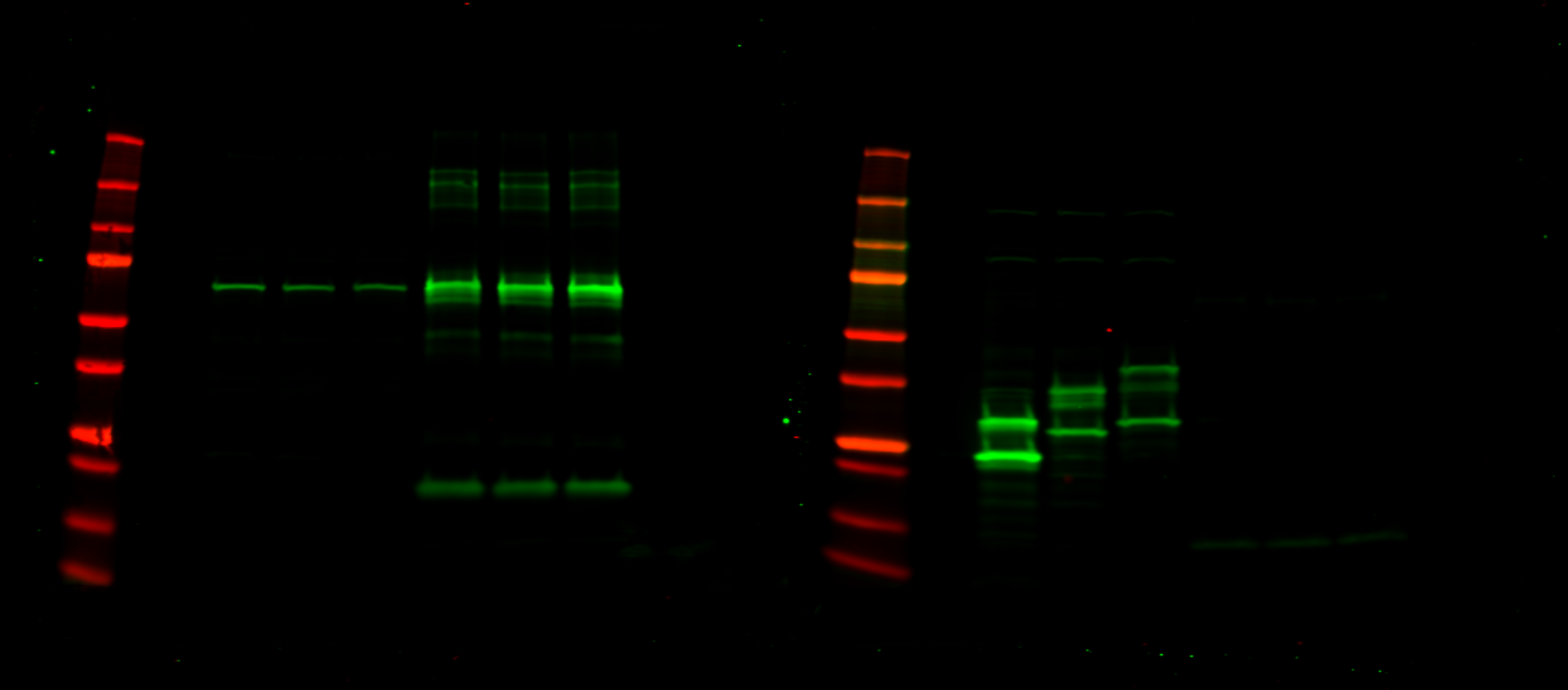

Supplement: Figure 9—figure supplement 3—source data 1. — Anti-GFP and anti-mCherry western blot from anti-GFP immunoprecipitation. [file elife-78653-fig9-figsupp3-data1.zip › Figure 9 - Figure Supplement 3 - Source Data 1/F9 FS3 IP Blot.tif]

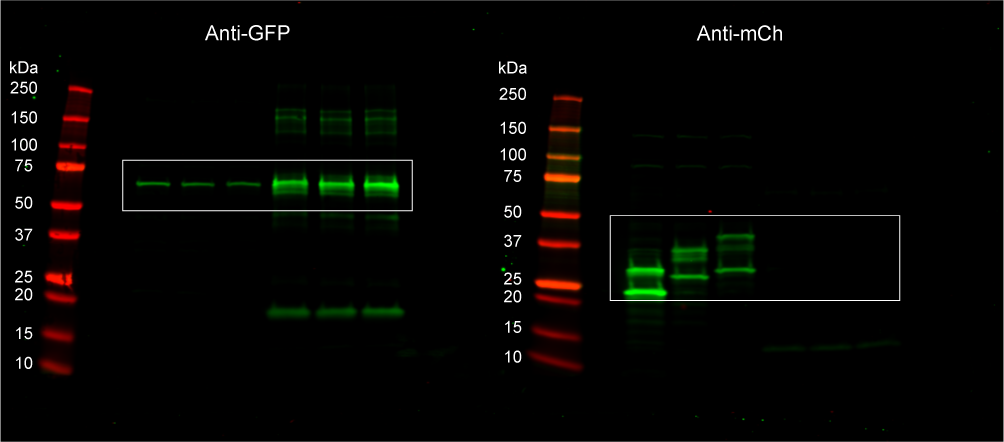

Supplement: Figure 9—figure supplement 3—source data 1. — Anti-GFP and anti-mCherry western blot from anti-GFP immunoprecipitation. [file elife-78653-fig9-figsupp3-data1.zip › Figure 9 - Figure Supplement 3 - Source Data 1/F9 FS3 IP Blot Annotated.tif]
